# Cohort Profile: A prospective cohort study of objective physical and cognitive capability and visual health in an ageing population of men and women in Norfolk (EPIC-Norfolk 3)

**DOI:** 10.1093/ije/dyt086

**Published:** 2013-06-14

**Authors:** Shabina A Hayat, Robert Luben, Victoria L Keevil, Stephanie Moore, Nichola Dalzell, Amit Bhaniani, Anthony P Khawaja, Paul Foster, Carol Brayne, Nicholas J Wareham, Kay-Tee Khaw

**Affiliations:** ^1^Department of Public Health and Primary Care, University of Cambridge, Cambridge, UK, ^2^NIHR Biomedical Research Centre at Moorfields Eye Hospital and University College London, London, UK, ^3^Department of Public Health and Primary Care, Institute of Public Health, University of Cambridge, Cambridge, UK and ^4^MRC Epidemiology Unit, Institute of Metabolic Science, Addenbrooke’s Hospital, Cambridge, UK

## Abstract

The European Prospective Investigation of Cancer (EPIC) is a 10-country collaborative study in which EPIC-Norfolk is one of the UK centres. EPIC-Norfolk examined 25 639 men and women resident in East Anglia (aged 40–79 years), between 1993 and 1997. The EPIC collaboration was set up to examine the dietary determinants of cancer, but the remit in the EPIC-Norfolk cohort was broadened from the outset to include determinants of other health conditions and chronic diseases. EPIC-Norfolk completed a third round of health examinations (EPIC-Norfolk 3 or 3HC) in December 2011, on 8623 participants in the age range 48–92 years. EPIC-Norfolk focused on objective measures of cognitive function, physical capability and visual health, adapting this existing mid-life cohort to the current need to investigate healthy and independent living for ageing societies. With a wealth of longitudinal data and a biobank (including DNA) collected at up to three separate time points, EPIC-Norfolk offers the unique opportunity to investigate the association of lifestyle and biological factors, including genetic exposures, with a range of health outcomes in middle and later life. Information for data access can be found on the study website, details as given in this cohort profile.

## Background

The numbers of older people worldwide are increasing at an unprecedented rate and good health and well-being in later life are now major priorities.^1^ To maintain good health in later life, we need to improve our understanding of how to influence conditions such as dementia, sarcopenia, age-related macular degeneration and glaucoma. These conditions are more prevalent in older age but are not necessarily an inevitable consequence of ageing, with substantial variations seen in the health and functional status of older people. Understanding determinants of these conditions may help us understand how best to postpone or reduce disability and disease in later life.

The occurrence of chronic disease, disability and dependence increases with age. With more people living longer, this is a challenge to society with considerable cost implications for our health services.^2^ Ageing is often characterized by a complex combination of morbidities, and we need to consider not just disease prevention but also slowing down the progression to disability. Delaying onset of disease and improving survival (e.g. through effective control of vascular risk factors) can reduce the numbers of individuals with disability in later life.^3^ A small change in the incidence of common conditions may have a profound impact on projections of future numbers with disability in the population.^3–6^ Through EPIC-Norfolk, we aim to get a better understanding of the causes of disease and the reasons for health inequalities so as to eventually inform policies for health improvement and disease prevention in middle and later life.

## Why was the EPIC-Norfolk cohort set up and what was the rationale for EPIC-Norfolk 3?

The European Prospective Investigation into Cancer (EPIC) is an international collaboration studying diet and disease, with half a million participants.^7^^,^^8^ EPIC-Norfolk is one of the UK centres of this 10-country collaboration and, although part of the diet and cancer study, its remit was widened soon after its inception to include investigation of major determinants of chronic disease, disability and death in middle and later life. EPIC-Norfolk has had a particular focus on characterizing exposures in terms of modifiable lifestyle factors such as diet, physical activity and psychosocial factors. Key priorities have been to develop improved methods of exposure measurement and to characterize participants extensively in terms of their lifestyle, physiological, metabolic and genetic profiles. A wealth of prospective data on health and lifestyle exposures including objective measures has now been collected over almost 20 years of follow-up.

The baseline health examination (1HC 1993–97) focused on obtaining detailed information on lifestyle (including diet and physical activity), medical history and measurements of cardiovascular disease risk factors including anthropometry and blood pressure. Characteristics of the cohort at baseline have been described previously.^9^ A second health examination (2HC 1997–2000) repeated measures collected at baseline, with the addition of heel bone ultrasound^10^ and impedance for body fat percentage.

The main focus of the third health examination, EPIC-Norfolk 3 (3HC 2006–11) was to investigate conditions relevant to ageing in participants now in the age range of 48–92 years. Measures taken previously were repeated to document longitudinal trajectories in behaviours, physiological characteristics and health status. EPIC-Norfolk 3 is centred mainly on three areas associated with major causes of loss of independence and increasing disabilities in later life: cognitive decline, loss of mobility and loss of vision, all which have been associated with the onset of disability.^11^^,^^12^

Although memory impairment and cognitive decline generally increase with age, there is a broad range of cognitive capability within the older population.^13^ Decline in muscle mass and function with advanced age (sarcopenia) is associated with considerable morbidity and mortality amongst older persons, with substantial healthcare costs.^14^ Visual impairment is not only a source of morbidity in itself, but also increases the risk of future functional impairment, disability and mortality.^15^ Certification figures for visual impairment for England and Wales show that the three leading causes for both blindness and partial sight are age-related macular degeneration, glaucoma and diabetic retinopathy.^16^ These eye conditions, along with uncorrected refractive error which [according to the World Health Organization (WHO)^17^] is globally the major cause of visual impairment, are the main ophthalmic conditions investigated in EPIC-Norfolk 3.

Procedures in this study were approved by the Norfolk Local Research Ethics Committee (05/Q0101/191) and East Norfolk and Waveney NHS Research Governance Committee (2005EC07L). Participants gave signed informed consent. As part of the research process, the EPIC-Norfolk team actively promotes the involvement of participants. A participants’ advisory panel (EPAP: EPIC Participant Advisory Panel) advises on various aspects of the study and gives their views on potential future projects.

## Who is in EPIC-Norfolk 3 and how often have they been followed up.

In total 77 630 individuals from 35 general practices in Norfolk were invited at baseline to take part in this prospective cohort study. Of these, 30 445 consented to take part and completed a health questionnaire. It is difficult to compare characteristics between responders and non-responders as limited data are available on non-responders. Of those approached, 42% of all women agreed to take part as compared to 36% of all men. Table 1 shows the distribution of participants, where non-responders were likely to be men and slightly younger. However, all ages were well represented for both men and women in the cohort including those in the 70+ age group. Also, as described previously, the participating cohort was similar to the national population samples studied in the Health Survey of England, in terms of anthropometry, serum lipids and blood pressure.^9^

**Table 1.** Age and sex of responders and non-responders at baseline

Variable	Responders	Non responders
	39% (*N* = 30 445)	61% (*N* = 47 185)
**Men** 48.7% (37 825)		
% (*N)*	36.2 (13 700)	63.8 (24 125)
Age group (years)		
≤44	4.3 (587)	6.5 (1572)
45-49	16.7 (2287)	21.0 (5061)
50-54	15.9 (2178)	18.2 (4385)
55-59	15.2 (2075)	15.1 (3649)
60-64	15.6 (2142)	13.1 (3165)
65-69	15.4 (2112)	11.8 (2848)
≥70	16.9 (2319)	14.3 (3445)
**Women** 51.3% (39 805)		
% (*N*)	42.1 (16 745)	57.9 (23 060)
Age group (years)		
≤44	4.6 (778)	6.2 (1422)
45-49	18.4 (3084)	18.7 (4304)
50-54	16.1 (2699)	16.1 (3717)
55-59	15.1 (2523)	13.8 (3189)
60-64	14.8 (2482)	13.0 (3009)
65-69	14.9 (2492)	13.4 (3080)
≥70 years	16.1 (2687)	18.8 (4339)

**Figure 1.**
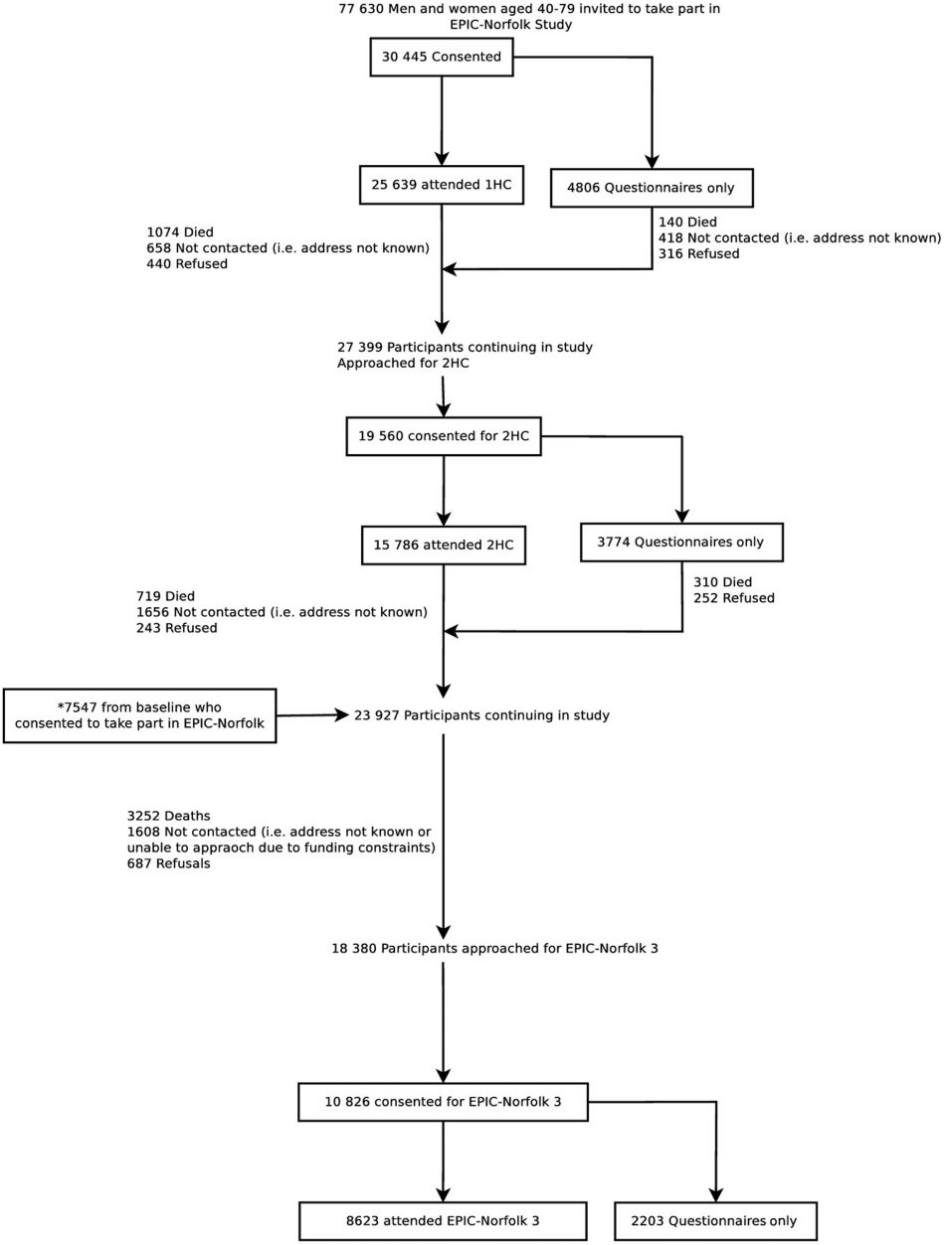
EPIC-Norfolk study over more than 18 years of follow up, showing numbers of participants who responded positively and attended health examinations and those lost to follow up at each phase *These 7547 participants consented to take part at baseline (of which 5970 attended 1HC), but did not attend 2HC, They are included in the count for participants remaining in Study at the time of EPIC-Norfolk 3 approach

Figure 1 summarizes the numbers of participants involved over the 19-year follow-up, and participation at each stage. In addition to health examinations, participants have been invited to complete health and lifestyle questionnaires at regular intervals. The number of participants who attended the 1HC was 25 639 (response rate of 33%). For subsequent approaches, all those who consented at baseline were invited to take part after excluding those who had died and those who had previously requested no further approaches. Record linkage to the NHS Exeter System ensured participant contact information was up to date. The number of participants attending the 2HC was 15 786 (a response rate of 57.6%), with the remainder (3774 participants) completing the health questionnaire only. The number of participants who died before the point in time when they were due to receive an invitation to 2HC was 1214. A further 1832 either refused or could not be contacted (i.e. address not known). Using the same criteria from the 2HC (inviting participants still alive, not having refused further approaches and with a contact address), 18 380 participants were approached for EPIC-Norfolk 3 (3HC) and the pilot phase. This included 7547 participants who did not attend the 2HC (but 79% of whom did attend the 1HC, the remaining 21% not attending the health examination, but completing a health and lifestyle questionnaire at baseline). There were 5495 deaths recorded up to this point. Of those invited to EPIC-Norfolk 3, 8623 (response rate of 46.9%) attended the health examination.

Participants were sent an invitation and a health and lifestyle questionnaire (HLQ) for EPIC-Norfolk 3. Participants sent their responses on a participation form indicating their preference of timings and returning their completed HLQ, in a freepost envelope. Every endeavour was made to facilitate participation (see Supplementary Appendix available as Supplementary data at *IJE* online). Practices were approached two at a time, based on geographical location and distance from the clinic in Norwich, i.e. one at close proximity (city practice) and one further afield (rural area). Funding constraints led to the exclusion of four practices from follow-up. As a result of not including these practices, 1517 eligible participants were not approached. The follow-up period between 2HC and the most recent phase was up to 10 years (compared with follow-up between 1HC and 2HC, which was up to 5 years).

To examine attrition in the cohort, the baseline characteristics of those who attended both the 1HC and EPIC-Norfolk 3 (3HC) were then compared with the 17 789 participants who attended the 1HC only (Table 2). The proportions of men and women were similar, with women comprising 55.3% of the group attending both baseline and follow-up health examinations and 54.5% of those attending the baseline health examination only (*P* = 0.2).

**Table 2.** Baseline characteristics of participants in EPIC-Norfolk. Participants who attended health examinations in both the first and third phases of EPIC-Norfolk (1HC + 3HC) are compared with those who were examined in the first phase only (1HC only)

Variable	Men			Women		
	1HC+ 3HC	1HC only	*P*-value	1HC+ 3HC	1HC only	*P*-value
	(*N* = 3615)	(*N* = 7992)		(*N* = 4495)	(*N* = 9537)	
Mean (SD)^a^						
Age (years)	56.6 (7.9)	61.0 (9.5)	<0.001	55.2 (7.8)	60.7 (9.5)	<0.001
Height (cm)	174.9 (6.5)	173.6 (6.7)	<0.001	162.1 (6.0)	160.4 (6.3)	<0.001
Body mass index (kg/m^2^)	26.1 (3.0)	26.7 (3.4)	<0.001	25.5 (4.1)	26.6 (4.4)	<0.001
Systolic blood pressure (mmHg)	134.2 (16.3)	138.9 (18.1)	<0.001	129.4 (17.1)	136.0 (19.4)	<0.001
Total cholesterol (mmol/l)	6.0 (1.1)	6.1 (1.1)	0.007	6.1 (1.1)	6.4 (1.2)	<0.001
Frequency, % (*N*)^b^						
Education						
No qualification	22.0 (795)	34.3 (2739)	<0.001	29.5 (1327)	48.2 (4593)	<0.001
O-level or equivalent (or above)	78.0 (2819)	65.7 (5245)	<0.001	70.5 (3167)	51.8 (4936)	<0.001
Social class (% I-IIINM)	65.0 (2333)	55.4 (4324)	<0.001	67.5 (3001)	58.5 (5393)	<0.001
Smoking						
Current	8.7 (313)	13.8 (1092)		8.8 (392)	12.6 (1187)	
Ex-smoker	49.7 (1792)	56.7 (4492)		30.3 (1354)	33.2 (3123)	
Never	41.6 (1498)	29.5 (2339)	<0.001	61.0 (2729)	54.2 (5108)	<0.001
Physical activity						
Inactive	23.1 (834)	34.4 (2752)		20.3 (914)	35.3 (3363)	
Moderately-Inactive	26.1 (945)	24.0 (1913)		33.2 (1493)	31.5 (3000)	
Moderately-active	25.0 (905)	22.0 (1755)		26.0 (1168)	20.4 (1948)	
Active	25.8 (931)	19.7 (1571)	<0.001	20.5 (920)	12.9 (1226)	<0.001
Median (IQR)^c^						
Alcohol intake (units/week)	7 (2.5, 14.5)	6 (2, 14)	<0.001	2.5 (1, 7.5)	2 (0.5, 6)	<0.001

Groups were compared using unpaired Student’s *t-*test^a^, chi square^b^ and Mann-Whitney^c^ tests as appropriate. 1HC, first health examination; 3HC, third health examination (EPIC-Norfolk 3); SD, standard deviation; IQR, interquartile range; I-IIINM: social class I-III Non-Manual.

Those who returned to take part in EPIC-Norfolk 3 were, at the time of the first health examination, more likely to be younger and taller and have lower weight, lower blood pressure and lower cholesterol concentrations. They were also more likely to be educated to at least to O-level standard or equivalent (i.e. leaving school with exams at 16 years of age), to have a higher socioeconomic status, to havenever smoked and to have been more physically active. Responders to EPIC-Norfolk 3 were also more likely to drink more alcohol at baseline than those who did not respond.

Table 2 shows that the trends seen in the baseline characteristics were the same in both men and women. Although, as might be expected, those attending EPIC-Norfolk 3 were younger, with a better cardiovascular disease risk profile and socioeconomic status at baseline compared with those not attending, the cohort still represents a diverse population with a wide socioeconomic distribution and range of lifestyle factors of interest, such as physical activity and obesity.

## What has been measured?

The 1HC and 2HC lasted approximately 30 min and comprised anthropometry, simple physiological measures including respiratory function [FEV1 (forced expiratory volume in 1 s)], blood pressure, venepuncture for blood and urine sample collection*.* In contrast, the EPIC-Norfolk 3 health examination took from 2 h 30 min to 3 h to complete, depending on the participant. Assessments were carried out using standardized procedures by nurses, who received an initial 3-month intensive training with annual refresher courses.

Participants taking part in EPIC-Norfolk 3 gave signed informed consent again to cover the new areas of data collection. A sample copy of the consent form was sent in the invitation pack for information, but consent was taken in the presence of the nurse (see the Supplementary App for details, available as Supplementary data at *IJE* online). Full lists of measurements, including repeated measures, are detailed in Boxes 1 and 2.

**Box 1 Self report data collected from questionnaires in EPIC-Norfolk 3 (details on these measures can be found in Supplementary Data)**

**Table:** 

**Health and Lifestyle Questionnaire (Follow-Up IV)**
Socio-demographic Employment status Self-rated health and diagnosis (including vision and hearing) Social networks and support, leisure activities and hobbies Activities of daily living Falls Medication Smoking and alcohol^a^ (Alcohol intake measure of unit per week was calculated from number of drinks consumed per day over 7 days, which was different from baseline which was calculated from total number of drinks consumed over 7 days). Self-perceived wealth and economic status^a^
**Health and Life Experiences Questionnaire (HLEQ)**
Psychosocial measures Widespread pain using the Manchester Coding System^a^ Social life Loneliness Anxiety and depression Mood status Health Daily activities, lifetime events Childhood experiences, personal beliefs
**Physical Activity Questionnaire (EPAQ2) and perception of local environment**
Self-report on physical activity behaviours in three domains: activity at home, work and recreation. Also, using Geographical Information Systems (GIS)^a^ with Neighbourhood Environment Walkability Scale (NEWS) to observe how environmental factors play a role in determining behaviour
**Skin ageing**
Self-report on exposure to UV sunlight (lifetime and previous year) Skin reaction to sunlight exposure Tanning (including attitude towards UV exposure) Use of sun protection/skin care Natural hair colour (at age 20 years and current)
**Dietary data**
7-day food diary and Food Frequency Questionnaire (FFQ)

^a^New measures in EPIC-Norfolk 3 (not applied at previous phases).

**Box 2 New and repeat objective measures applied at clinic**

**Table:** 

Venous blood sample	Biomarkers included full blood count (platelets, total white blood count, neutrophils, basophils, eosinophils, monocytes, lymphocytes, total red blood cell count), mean corpuscular volume, hamatocrit, haemoglobin; glycated haemoglobin; lipid profile (total cholesterol, HDL LDL, triglyceride); vitamin C; creatinine; albumin; and C-reactive protein. Serum, plasma and whole blood also stored for future biochemical profiling and DNA extraction
Anthropometric measures	Standing height (Stadiometer, Chasmores, UK), weight, waist and hip circumference
Impedance/body fat	Body fat percentage measured using TANITA TBF-300 MA Body Composition Analyser (Tanita UK, Yiewsley, UK)
Physiological functions	Brachial pressure and heart rate measured with Accutorr PlusTM automatic sphygmomanometer blood pressure monitor (Datascope Medical, Huntingdon, UK). Also measured was Ankle Brachial Pressures^a^using the mini Dopplex D990 Doppler Pen with ultrasonic Doppler flow detector (Huntleigh Healthcare, UK) and respiratory function using a portable spirometer (Micro Medical, UK).
Ultrasound measurements of the calcaneus	Attenuation of broadband ultrasound(dB/MHz) and speed of sound (m/s) were measured three times on each foot with CUBA clinical instrument (McCue Ultrasonics, Winchester, UK)
Skin ageing^a^	Digital Images of skin on face and hands taken and stored for future grading
Cognitive assessmen^a^	Retrospective and prospective memory, attention and calculation, registration, new learning, language, executive function, proxy measure of IQ and visuospatial /constructional ability
Physical capability^a^	Usual walking speed, standing balance, chair stands, grip strength using a Smedley’s Dynamometer (Scandidact, Kvistgaard, Denmark)
Objective measure of physical activity^a^	Physical activity using a commercial accelerometer, the GT1M (Actigraph, Florida, USA)
Eye examination^a^	Visual acuity using the LogMAR visual acuity chart 1 (Precision Vision, LaSalle, IL, USA), intraocular pressure using an AT555 Non-Contact Tonometer (Reichert, New York, USA) and later using the Ocular Response Analyzer (ORA, Reichert, New York, USA), axial length and anterior chamber depth using IOLMaster, (Carl Zeiss Meditech, Welwyn Garden City, UK), retinal nerve fibre layer thickness (GDx VCC, Zeiss, Dublin, CA, USA). Threshold visual field analysis was done with the Humphrey field analyser (Carl Zeiss Meditech), optic nerve head topography determined using the HRT II (Heidelberg Retina Tomograph, Heidelberg Engineering, Heidelberg, Germany), colour fundus photography of optic disc and macula using a Topcon non-mydriatic retinal camera TRC-NW6S and IMAGEnet Telemedicine System (Topcon Corporation, Tokyo, Japan) with a 10- megapixel Nikon D80 camera (Nikon Corporation, Tokyo, Japan).
Medication	Confirmation of medication by nurse using repeat prescription slips

^a^New measures in EPIC-Norfolk 3 (not applied at previous phases).

At the end of the health examination, participants were given a feedback form (which was optional and anonymized, unless the participant chose to give their contact details) to gather information on the quality of the experience of the health examination. Participants were given feedback on some results and clinically relevant results were sent to the general practitioners (with the permission of participants) with pre-agreed threshold levels for immediate notification. The data from the eye examination were reviewed by an ophthalmologist and participants with abnormal results were referred to a specialist clinic set up at the Norfolk & Norwich University Hospitals (NNUH) NHS Foundation Trust.

## What has it found? Key findings and publications

EPIC-Norfolk has published several hundred papers using data collected prior to EPIC-Norfolk 3. These can be found on our website (www.epic-norfolk.org.uk). Included in our key findings are some high profile papers such as those showing that the combined impact of four health behaviours (not smoking, drinking between 1 and 14 units of alcohol, having some daily physical activity and eating at least five portions of fruit and vegetables every day) resulted in lower cardiovascular and mortality rates. Furthermore, those with all four behaviours each day lived on average 14 years longer than people who adopted none.^18^ This finding was used to inform the Government’s *Small Change, Big Difference Campaign* in 2006.

The data collection phase for EPIC-Norfolk 3 ended in December 2011, and the data are currently being cleaned and made available for further analysis. Five papers (listed below) have been published using interim data. A key finding is that although the cohort is somewhat selected at this stage of the study, and prevalence of severe impairment is relatively low in our participants, there is considerable heterogeneity in function and health in the EPIC-Norfolk 3 cohort (Table 3), with some participants in the oldest age group performing better or with better function and health than those in the youngest group.

**Table 3.** Range of function and health observed in the EPIC-Norfolk 3 cohort

Variables		*N*	Mean (SD)	Median (IQR)
**Men (*N* = 3861)**	Systolic blood pressure (mmHg)	3860	136.4 (15.4)	136.5 (126.5, 146.5)
	Total cholesterol (mmol/l)	3604	5.0 (1.1)	4.9 (4.2, 5.7)
	Body mass index (kg/m^2^)	3850	27.1 (3.6)	26.7 (24.7, 29.0)
	Derived full MMSE score	3625	27.5 (1.4)	28.0 (27.0, 29.0)
	Grip strength of strongest hand (kg)	3812	39.1 (8.3)	39.0 (33.5, 45.0)
	Intraocular pressure (mm/Hg)	3753	16.2 (3.8)	15.6 (13.5, 18.4)
	Smoking, % (*N*)			
	Current	4.2 (159)
	Ex-Smoker	51.2 (1949)
	Never Smoker	44.6 (1695)
	Physical Activity, % (*N*)	
	Inactive	37.4 (1422)
	Moderately-inactive	25.0 (954)
	Moderately active	18.8 (713)
	Active	18.8 (714)
	Alcohol intake (units/week)			10.0 (2.0, 23.0)
**Women (*N* = 4762)**	Systolic blood pressure (mmHg)	4758	135.9 (17.1)	136.0 (124.5, 146.5)
	Total cholesterol (mmol/l)	4369	5.7 (1.1)	5.7 (5.0, 6.4)
	Body mass index (kg/m^2^)	4753	26.6 (4.8)	25.9 (23.3, 29.0)
	Derived full MMSE score	4333	27.5 (1.5)	28.0 (27.0, 29.0)
	Grip strength of strongest hand kg)	4661	24.3 (5.6)	24.5 (21.0, 28.0)
	Intraocular pressure (mm/Hg)	4640	16.3 (3.5)	16.1 (14.0, 18.4)
	Smoking, % (*N*)			
	Current	4.5 (213)
	Ex-Smoker	29.8 (1400)
	Never Smoker	65.7 (3085)
	Physical activity, % (*N*)	
	Inactive	37.2 (1748)
	Moderately-inactive	32.2 (1513)
	Moderately active	17.0 (796)
	Active	13.6 (641)
	Alcohol intake (units/week)			4.0 (0.0, 12.0)

SD, Standard Deviation; mmHg, millimetres of mercury; SF-MMSE, Short form MMSE Score; IOP, Intraocular pressure; IQR, Inter-quartile Range

### Validation of the short form mini-mental state examination (SF-MMSE)^19^

The SF-MMSE is a shortened version of the widely used Mini Mental State Exam (MMSE).^20^ We demonstrated that the SF-MMSE captures the full range of scorers from the severely impaired to the high functioning and that the full-scale MMSE scores could be accurately derived from this 11-item abbreviated version. This is important within frameworks such as epidemiological studies that require shorter testing methods but need results that are comparable to studies where the full test version has been administered.

### Physical activity and ocular perfusion pressure (OPP)^21^

OPP is the difference between arterial blood pressure (BP) and intraocular pressure (IOP), and low OPP has been implicated as a risk factor for open-angle glaucoma (OAG). Using eye data from EPIC-Norfolk 3 and physical activity data from an earlier phase has shown that individuals with a previous active lifestyle have a lower risk of a low OPP. Although further investigations are needed, these results show that physical activity may be a safe and simple way of reducing the risk of developing OAG.

### Refractive error (RE), axial length and anterior chamber depth of the eye^22^

Axial length of the eye is associated with RE, a major cause of visual impairment and disability in the UK, as well as a risk for glaucoma. Findings from EPIC-Norfolk 3 have added support to previous work which suggests that exposure to a lifestyle associated with greater near-work activity drives RE towards myopia (of which the mechanistic outcome is longer axial length), with individuals with higher education having longer axial length.

### Uncorrected refractive error (URE) in older British adults^23^

Uncorrected refractive error (URE), although low in the EPIC-Norfolk 3 cohort, was found to be associated with age, reduced ownership of spectacles or contact lenses for distance vision and in those who self- reported poorer distance vision. Being able to identify people with poor vision is important in planning provision of services to improve or correct vision.

### Intraocular pressure (IOP) and corneal biomechancs in older adults^24^

IOP is known as the main modifiable risk factor for glaucoma, but IOP may be prone to measurement error due to the biomechanical properties of other components of the eye, in particular the cornea. We attempted to obtain an accurate measure of IOP in EPIC-Norfolk 3, accounting for the biomechanics of the cornea. IOP was found to be higher in younger people, women, those with higher systolic blood pressure and those in sedentary occupation. We also found the biomechanical characteristics of the cornea, specifically the corneal resistance factor and corneal hysteresis, declined with age and were higher in women.

## What are the main strengths and weaknesses?

EPIC-Norfolk 3 provides objective measures of physical capability, cognitive function and visual health, including retinal imaging, on a very well-characterized cohort. A major strength of the EPIC-Norfolk study is the availability of longitudinal exposure and health outcome data from baseline (1993–97) to the present. From the 1HC onwards, emphasis has always been placed on using validated instruments and objective biomarkers to measure exposures including diet and physical activity. In particular it provides the opportunity to examine the trajectory of functioning in the general population and in particular the determinants of high as well as poor performance.

Most large cohort studies have to rely on linkage with death records to obtain mortality by cause, and on self- reported questionnaires for non-fatal health outcomes which are limited by response rates and subjective recall. The location of the cohort in a geographical region, Norfolk, means that follow-up through hospital record and disease register linkage and validation of medical records is facilitated, enabling identification of major non-fatal health endpoints such as strokes, heart disease, diabetes, fractures and arthritis in the whole cohort, in addition to mortality and cancer by cause.

The main limitations of the study are those that concern all cohort studies, in particular healthy volunteer bias and attrition. As one would expect, individuals would be less likely to participate in either the baseline or subsequent follow-up examinations if they were seriously ill, disabled or had major cognitive or visual impairment.^9^ In EPIC-Norfolk 3, there was likely to be selective truncation of individuals from the cohort at the lower end of the distribution of functional performance and some loss of frailer members of the cohort, but there remains a large range of performance and health to examine determinants of healthy ageing. Furthermore, the availability of baseline characteristics and mortality, and follow-up of the original cohort, enable characterization of both those who have and those who have not participated in follow-up examinations.

## Can I access of the data? Where can I find out more?

EPIC-Norfolk has a wide range of collaborators. Contact details, publications and the process for collaborating and data requests can be found on the website (www.epic-norfolk.org.uk). Requests are reviewed by the EPIC-Norfolk management committee and proposals should fulfil a number of criteria including that the work is within the bounds of consent given by participants. Scientific proposals must be satisfactorily peer-reviewed and ethically reviewed and approved.

## Supplementary Data

Supplementary data are available at *IJE* online.

## Funding

The infrastructure and core functions of this study are supported by the Medical Research Council, UK (G0401527) and Cancer Research UK (C864/A8257). The EPIC-Norfolk 3 clinic was funded by Research into Ageing (262). V.L.K. and A.P.K. are supported by Wellcome Trust research training fellowships.

## Supplementary Material

Supplementary Data
