# Supplementary material for: Cohort Profile: A prospective cohort study of objective physical and cognitive capability and visual health in an ageing population of men and women in Norfolk (EPIC-Norfolk 3)
Source: Int J Epidemiol. 2013 Jun 14;43(4):1063–72. doi: 10.1093/ije/dyt086 (PMC4121549; doi:10.1093/ije/dyt086)
Supplement: Supplementary Data [file supp_43_4_1063__index.html]

Cohort Profile: A prospective cohort study of objective physical and cognitive capability and visual health in an ageing population of men and women in Norfolk (EPIC-Norfolk 3) — Supplementary Data 

# Cohort Profile: A prospective cohort study of objective physical and cognitive capability and visual health in an ageing population of men and women in Norfolk (EPIC-Norfolk 3)

## 

files

**Files in this Data Supplement:**

- Supplementary Data - doc file
