# Supplementary material for: Cohort Profile: A prospective cohort study of objective physical and cognitive capability and visual health in an ageing population of men and women in Norfolk (EPIC-Norfolk 3)
Source: Int J Epidemiol. 2013 Jun 14;43(4):1063–72. doi: 10.1093/ije/dyt086 (PMC4121549; doi:10.1093/ije/dyt086)
Supplement: Supplementary Data [file supp_dyt086_dyt086_supplementary_data.doc]

SUPPLEMENTARY INFORMATION

**A prospective cohort study of objective physical and cognitive capability and visual health in an ageing population of men and women in Norfolk (EPIC-Norfolk 3)**

**Pilot**

The study was piloted using one of the collaborating GP practices for over one year in 2004. The results of the pilot gave an indication of the response rate for the health examination (or health check as referred to in EPIC-Norfolk). Although the pilot practice was not the furthest from the clinic which was based in Norwich, it showed that EPIC participants were willing to travel a significant distance (15 miles in this case) to take part. The pilot indicated the feasibility of including new measures as well as measures collected in previous examinations. The pilot was used to make modifications to the protocol where necessary, making some tests that were usually used in diagnostics, more suitable to an epidemiological setting. The pilot also demonstrated that with sufficient training, nurses (with no previous experience in ophthalmology) were able to conduct the eye examinations effectively.

**Recruitment and training of staff**

Nurses employed for this study were professionally qualified with experience of performing phlebotomy. Intensive 3 month training was provided initially. Nurses were given a logbook to complete during their training period, which was signed off by the Clinic Manager/Coordinator once the member of staff was conversant with a particular component of the health examination. Nurses were trained to follow the detailed standard operating procedures (SOPs) to ensure accuracy and consistency. These SOPs were reviewed and updated regularly. Refresher courses, updates and quality control sessions were given throughout the year and monthly progress meetings held between staff based in Norwich and Cambridge.

**Recruitment of Participants**

Thirty-five GP sites have been collaborating with EPIC-Norfolk since 1993. Practices were approached for EPIC-Norfolk 3 (two at a time) according to their geographical location and distance from the clinic, selecting one close proximity and one from a further distance. This was to ensure that all appointment slots could be filled, and that both urban and rural practices were approached simultaneously. Once the practice agreed to collaborate, EPIC participants were approached directly by the research team in Cambridge. Documentation sent to participants at the original approach included a participant information sheet, Health and Lifestyle Questionnaire, Food Frequency Questionnaire, sample of the consent form (to read and familiarise themselves, but not to complete before attending the health examination) and a participation form. Participants were also provided with a pre-paid envelope to return the completed documentation.

**Consent**

All EPIC participants have consented at baseline to participate in the long term study. However consent was taken again from participants taking part in EPIC-Norfolk 3 to cover the new procedures that did not fall within the existing consent and to update the consent to comply with current standards. In the invitation, participants were asked to read the information sheet relating to the study and were asked to complete a participation form indicating their willingness to take part and their preferred day and time for their appointment. They could also, at this point, refuse (and if they chose, give reasons for refusal). If the participant agreed to take part, they were asked to read the consent form carefully, but not complete it until they arrived at the clinic. Consent was then given in the presence of the nurse who explained the health examination to the participant, answered questions and ensured that the participant was completely informed of what they were being asked to do, before signing the consent form. It was also made clear to the participant that they could refuse any part of the health examination should they wish to do so.

**Questionnaires**

Health and Lifestyle

Health and lifestyle data were collected on the self-completion questionnaire, and included health variables (e.g. self-reported health, medications, physical performance, amount of physical activity, cognitive functioning, level of difficulties with activities of daily living and information regarding sexual function); psychological variables, (e.g. mental health, well-being, life satisfaction, mood); socio-economic variables (e.g. current work activity, reasons for retiring, financial security); and social activity (leisure activities).

Nutrition

Participants were sent a Food Frequency Questionnaire (FFQ) with their appointment letter which was returned when attending the health examination. A seven day food diary (7DFD) was also given to participants by the nurse at the health examination with instructions. Participants were asked to return this in a pre-paid envelope.

Skin Ageing

A short questionnaire on extrinsic factors (in particular chronic sun exposure as well as other environmental factors, such as use of tanning beds and types of sun protection used) was sent to participants prior to their appointment. This was also returned on the day of their health examination.

Other Questionnaires

During 2004-2008, participants completed a Health and Life Experiences Questionnaire (HLEQ2) which included repeat questions from the first psychosocial questionnaire HLEQ^1^ to give an assessment of social and psychological circumstances and mood status over the life course. New measures included ‘Widespread pain’, using the Manchester coding system, a system shown to be effective for use in epidemiological studies.^2,3^ This questionnaire was completed by 14 804 participants. Participants also completed two physical activity questionnaires to investigate the relationship between physical activity and the environment, both that perceived by individuals and what was actually present (the objective environment). The new questionnaire on the environment was developed using Geographical Information Systems (GIS) technology ^4^ to investigate the relationship between physical activity and the perceived and objective environment in order to observe how environmental factors play a role in determining behaviour. Participants were also asked to complete the EPIC-Norfolk Physical Activity Questionnaire (EPAQ2), a repeat questionnaire from the 2HC.^5^

**Physical Measures**

Skin Ageing

Digital photographs of the skin (face and hands) were taken under standardised conditions. These photographs will be used in the future to assess signs of skin ageing.

Cognition

Cognitive function was measured using nine previously validated tests assessing different cognitive domains to make up a comprehensive cognition battery (EPIC-COG). Cognitive tests used in EPIC-Norfolk 3 included the P&W Letter Cancellation Test; Assessment of prospective memory (memory for future actions);^6^ selected items from Cambridge Cognition Examination (CAMCOG) which forms part of the CAMDEX interview;^7,8^ the Extended Mental State Exam (EMSE) ^9^ and animal naming (verbal fluency), all which have been used in the Medical Research Council Cognitive Function and Ageing Study (MRC CFAS) ^10^. Also included was a shortened form of the original Mini Mental State Examination, the SF-MMSE ^11^ measuring memory, recall (immediate and delayed), attention, calculation, object naming, verbal registration, language and visuospatial/constructional performance; The Cambridge Neuropsychological Test Automated Battery Paired Associates Learning Test (CANTAB-PAL); ^12,13,14^ the Hopkins Verbal Learning Test (HVLT); ^15^ the Shortened National Adult Reading Test (NART)^16^ and the Visual Sensitivity Test (VST) ^17^ which assesses disturbance in the magnocellular pathway.

Physical Capability

Objective measures of muscle strength and physical performance are commonly used to assess physical capability, the ability to perform activities of daily living independently. The Established Populations for the Epidemiologic Studies of the Elderly (EPESE) battery ^18^ was modified for use in EPIC-Norfolk. Usual walking speed, timed chair stands and standing balance tests were assessed. Additionally, hand grip strength, a marker of general muscle strength, was measured in both hands (recording which is the dominant hand) using a hand-held dynamometer (Smedley’s Dynamometer, Scandidact, Kvistgaard, Denmark). Our protocol was based on that used in Survey of Health, Ageing and Retirement in Europe (SHARE), where participants were asked to squeeze the lever of the dynamometer as hard as possible for a few seconds with elbow at 90°, keeping the upper arm tight against the trunk, preferably whilst standing ^19^. The EPESE battery has been shown to demonstrate a range of function even amongst non-disabled older people and is associated with future disability, institutionalisation and death.^18,20^ Muscle strength ^21,22^  and individual performance measures such as walking speed ^23^ have also been strongly associated with future negative health outcomes ^24^ in a range of older populations.

Visual Health

Examinations were carried out according to Moorfields Eye Hospital standard operating procedures with regular training and validation. The eye exam consisted of nine tests. Imaging tools were used to acquire objective, precise and highly reproducible images of the eye. All tests were done without dilating the pupils. The measures included habitual and pinhole-corrected visual acuity measured using a LogMAR chart (log of minimum angle resolvable) at a distance of 4 metres using a standard ETDRS chart, employing an abbreviated testing protocol. Refraction was measured using the Humphrey model 500 autorefractor. Ocular axial dimensions (anterior chamber depth and axial length) and corneal radius of curvature were measured 5 times using partial coherence interferometry (Zeiss Humphrey IOL Master), which gives highly accurate, reproducible measurements. Intraocular pressure (IOP) measured three times in each eye by non-contact tonometry using the Reichert Ocular Response Analyser (ORA), which generated “Goldmann” equivalent intraocular pressure (IOPg), together with measures of ocular biomechanics including corneal hysteresis and resistance factor. These allowed the calculation of intraocular pressure adjusting for corneal biomechanics (IOPcc). Non-mydriatic digital photography of macula and optic disc (30° images) were taken using Topcon a TRC-NW6S. Assessment of retinal nerve-fibre layer thickness was measured using scanning laser polarimetry with variable corneal compensation (GDx-VCC). Optic disc surface topography was assessed using scanning laser ophthalmoscopy (HRT II, Heidelberg Engineering, Germany). In a small number of participants, cross-sectional anterior segment imaging and anterior chamber depth measurement were performed with anterior segment slit-lamp mounted optical coherence tomograph (SL-OCT Heidelberg Engineering, Heidelberg, Germany). On-going data review was carried out by a consultant ophthalmologist. Those with abnormal results were referred to the specialist clinic set up at the Norfolk & Norwich University Hospitals [NNUH] NHS Foundation Trust, for further assessment

Repeat Measures and additional other measures

Measures from the previous two health examinations were repeated to enable assessment of longitudinal changes. Anthropometric measurements were taken with participants in light clothing without shoes. Standing height was measured using a portable stadiometer, waist, hip and chest circumference was measured using D-loop tape measure, all to the nearest 0.1cm. Two measurements were taken and if the readings differed by more than 3 cm, then a third reading was taken. Weight and impedance were measured using TANITA TBF-300 MA Body Composition Analyser. Peak Expiratory Flow (PEF), Forced Vital Capacity (FVC) and Forced Expired Volume in one second (FEV1) were assessed using Micro plus hand held Spirometer (Micro Medical Instruments) to give a measure of lung function. Blood pressure was measured in the dominant arm with the participant seated, and the arm resting comfortably using the Accutorr Plus TM automatic sphygmomanometer (Data scope, UK). Two measurements were taken during the course of the health examination. Bone density was measured using the calcaneus (heel bone), which is known to be sensitive to osteoporotic change, using the McCue CUBA sonometer. The quantitative ultrasound (QUS) of the heel bone was measured (three times in each foot) as broadband ultrasound attenuation (BUA; in decibels per megahertz) and speed of sound (VOS).

In addition, new measurements were added to complement previous measurements. This included ankle brachial pressures to obtain the ratio of the ankle to brachial systolic blood pressure (the Ankle Brachial Index or ABI), as an addition to the conventional cardiovascular risk factors already measured. Participants rested for a minimum of five minutes in the supine position prior to blood pressure measurement. Two systolic blood pressure measures were taken using an 8 megahertz Doppler pen probe and an ultrasonic Doppler flow detector (Huntleigh Healthcare Ltd, Cardiff, UK.) from each limb on the right side only. If the repeat blood pressure differed by more than 10 mmHg at that site, then a third reading was taken. The cuff was inflated quickly to the maximal inflation level (until the pulse was no longer audible) and then deflated at a rate of 2 mmHg per second until the systolic blood pressure became audible. The cuff was applied to the bare ankle with the midpoint of the bladder over the posterior tibial artery approximately three centimetres above the medial malleolus.

Also included were the objective measures of physical activity using a commercial accelerometer, the GT1M (Actigraph, Florida, USA) worn by the participant for 7 days during waking hours.

**Data Capture**

All data were entered directly into an electronic case report form (eCRF) by the nurse. Each participant was assigned a new unique identifier bio-id number (which differed from the EPIC Study identifier given to each participant at baseline entry to the study). Hard copies of the CRF generated at the end of each appointment, plus pen and paper test data (such as those from the cognitive tests) were also attached.

Data checking (for accuracy and completeness), was a three-staged process, with the nurse entering the data documentation at the end of the appointment, first checking that most issues involving missing data were corrected immediately. The second stage involved a second member of staff, checking for missing data and the third was carried out on an ad hoc basis by the clinic manager. All amendments were clearly noted and any trends observed were relayed to the nurse involved and re-training carried out where necessary. Hard copies of the CRF and electronic data were transferred to Cambridge on a regular basis using encrypted portable hard drives. These were wiped, once data was removed.

**Results and Referrals**

Clinical results were made available to general practices on a monthly basis as a consolidated report via a secure electronic document transfer system. Results outside national recommended reference ranges were highlighted. EPIC-Norfolk thresholds were extreme values and practices were notified of these results. The data from the eye examination was reviewed by an ophthalmologist (PF, JY or AK) and participants with abnormal results were referred to a specialist clinic set up for EPIC-Norfolk participants at the Norfolk and Norwich University Hospital.

The average broadband attenuation of sound waves (BUA) for heel bone measure was compared to the general EPIC-Norfolk population tested during the second health examination (2HC 1997-2000) matched by sex and age-group. A mean BUA value (of 2 or more standard deviations) below the mean BUA reading of someone of the same sex and similar age was flagged to the general practitioner in the monthly report as someone who might warrant further investigations.

**References**

1. Surtees, P. G., Wainwright, N. W. J., Khaw, K.-T., & Day, N. E. (2003). Functional health status, chronic medical conditions and disorders of mood. British Journal of Psychiatry, 183, 299–303.
2. MacFarlane GJ, Croft PR, Schollum J, et al. Widespread pain: is an improved classification possible? J Rheumatol 1996;23:1628–32
3. Hunt IM, Silman AJ, Benjamin S, et al. The prevalence and associated features of chronic widespread pain in the community using the ‘Manchester’ definition of chronic widespread pain. Rheumatology (Oxford) 1999;38:275–9
4. Saelens BE, Sallis JF, Black JB, Chen D. Neighborhood-based differences in physical activity: an environment scale evaluation. Am J Public Health 2003;93(9):1552-8.
5. Wareham NJ, Jakes RW, Rennie KL, Mitchell J, Hennings S, Day NE. Validity and repeatability of the EPIC-Norfolk physical activity questionnaire. Int J Epidemiol 2002; 31:168 – 74.
6. Huppert FA, Johnson AL, Nickson J, MRC CFAS: High prevalence of Prospective Memory Impairment in the Elderly and in Early-stage Dementia: Findings from a Population-based Study. Applied Cognitive Psychology 2000, 14: S63-S81
7. Roth M, Tym E, Mountjoy CQ, Huppert FA, Hendrie H, Verma S, and Goddard R: CAMDEX. A standardised instrument for the diagnosis of mental disorder in the elderly with special reference to the early detection of dementia. The British Journal of Psychiatry 1986 149: 698-709.
8. Roth M, Huppert FA, Tym E, Mountjoy CQ: CAMDEX: The Cambridge Examination for Mental Disoders of the Elderly. Cambridge, Cambridge University Press; 1988

9. Huppert FA, Cabelli ST, Matthews FE, and the MRC Cognitive Function and Ageing Study (MRC CFAS): Brief cognitive assessment in a UK population sample – distributional properties and the relationship between the MMSE and an extended mental state examination. BMC Geriatrics 2005; 5:7

1. MRC CFAS: Cognitive function and dementia in six areas of England and Wales: The

distribution of MMSE and GMS organicity level in the MRC CFA Study. The Medical Research Council Cognitive Function and Ageing Study (MRC CFAS). Psychol Med 1998, 28: 319-335

1. Matthews FE, Stephan B.C.M, Khaw K T, Hayat S, Luben R, Bhaniani A, Moore S, Brayne C: Full-scale scores of the Mini Mental State Examination can be generated from an abbreviated version. J Clin Epidemiol 2011 64(9): 1005-13 Epub 2011 Mar 16
2. Sahakian B, Morris R, Evenden J, Heald A, Levy R, Philpot M and Robbins T (1988). A comparative study of visuospatial memory and learning in Alzheimer-type dementia and Parkinson’s disease. Brain 111, 695-718
3. Fowler KS, Saling MM, Conway EL, Semple JM, Louis WJ: Paired Associates Performance in the early detection of DAT. J Int Neuropsychol Soc 2002 8(1) 58-71
4. Fowler KS, Saling MM, Conway EL, Semple JM, Louis WJ: Computerized neuropsychological tests in the early detection of dementia. J Int Neuropsychol Soc 2002 3 139-146
5. Brandt J. (1991). The Hopkins Verbal Learning Test: Development of a new memory test with six equivalent forms. The Clinical Neuropsychologist, 5, 125-142

16. Beardsall, L. & Brayne, C. Estimation of verbal intelligence in an elderly community: A prediction analysis using a shortened NART. British journal of Clinical Psychology, 1990 29, 83-90

17. Kirby, L., Bandelow, S, Hogervorst, E. (2010) Visual impairment in Alzheimer’s disease Journal of Alzheimer’s disease Feb 24 epub

18. Guralnik JM, Simonsick EM, Ferrucci L et al. A short physical performance battery assessing lower extremity function: Association with self-reported disability and prediction of mortality and nursing home placement. J Gerontol 1994;49:M85–M94

19. Andersen-Ranberg, K., Petersen, I., Frederiksen, H., Mackenbach, J. P., & Christensen, K. (2009). Cross-national differences in grip strength among 50+ year-old Europeans: results from the SHARE study. European Journal of Ageing, 6(3), 227–236.

20. Guralnik JM, Ferrucci L, Simonsick EM et al. Lower-extremity function in persons over the age of 70 years as a predictor of subsequent disability. N Engl J Med 1995;332:556–561

21. Sayer, A. A., Syddall, H. E., Martin, H. J., Dennison, E., Anderson, F. H., & Cooper, C. (2006). Falls, sarcopenia, and growth in early life: findings from the Hertfordshire Cohort Study. American Journal of Epidemiology, 164(7), 665– 671.22.

22. Ruiz, J. R., Sui, X., Lobelo, F., Morrow, J. R., Jackson, a. W., Sjostrom, M., & Blair, S. N. (2008). Association between muscular strength and mortality in men: prospective cohort study. BMJ, 337 a439

23. Elbaz, A., Sabia, S., Brunner, E., Shipley, M., Marmot, M., Kivimaki, M., & Singh-Manoux, A. (2012). Association of walking speed in late midlife with mortality: results from the Whitehall II cohort study. Age (Dordrecht, Netherlands) .DOI 10.1007/s11357-012-9387-9.

24. Cesari, M., Kritchevsky, S. B., Newman, A. B., Simonsick, E. M., Harris, T. B., Penninx, B. W., Brach, J. S., et al. (2009). Added value of physical performance measures in predicting adverse health-related events: results from the Health, Aging And Body Composition Study. Journal of the American Geriatrics Society, 57(2), 251–9.
